# *In vitro* antioxidant and antinociceptive properties of *Porphyra vietnamensis*

**DOI:** 10.1051/bmdcn/2019090103

**Published:** 2019-02-22

**Authors:** Saurabh Bhatia, Satish Sardana, Kishan Ram Senwar, Anjali Dhillon, Ajay Sharma, Tanveer Naved

**Affiliations:** 1 Amity Institute of Pharmacy, Amity University Haryana Gurgaon-122413 India; 2 Amity Institute of Pharmacy, Amity Institute of Pharmacy, Amity University Madhya Pradesh Maharajpura Gwalior (MP)-474005; 3 Amity Institute of Pharmacy, Amity University Noida Uttar Pradesh-201313 India

**Keywords:** Porphyra, Antioxidant, Antinociceptive, Extract, Red alga, Analgesic

## Abstract

Background: Current investigation explores the anti-oxidant and antinociceptive potential of edible seaweed namely Porphyra vietnamensis.

Methods: Radical scavenging and antinociceptive potential of ethanolic (EE), aqueous (AE), acetone (ACE) and chloroform (CE) fractions were determined using various models and assays. Writhing, formalin, hot plate, acetic acid induce response models were performed to determine antinociceptive activity whereas different assays have been used to determine antioxidant potential.

Results: Among the various fractions, ACE showed maximum biological activity. In DPPH assay half maximal inhibitory concentration (IC50) was found to be 0.470 ug/ml (DPPH assay), 0.381 jug/m/ (H2O2 assay), 0.470 ug/ml (super oxide assay), 0.591ug/ml (lipid peroxidation) and 0.430 ug/ml (nitric oxide assay). However, comparatively the TPC was more in EE (977.0 mg GAE/gm DW).

Conclusion: It was concluded that acetone fraction of Porphyra showed marked antinociceptive and antioxidant activities, however pharmacological and chemical investigations are required to identify principle compounds responsible for activities and characterize their respective mechanism(s) for respective actions.

## 1. Introduction

Marine environment offers 80% of world’s species of flora and fauna [1]. From several decades humans are exploiting marine resources especially seaweeds as food sources [2]. These resources represent a potential source of therapeutic compounds especially secondary metabolites with diverse pharmacological properties which can be utilized for the prevention or treatment of several disorders [3]. From the past few decades discovery of metabolites with potential biological activities from marine resources has increased considerably [4]. Recently seaweeds have received considerable attention from several scientific researchers [5]. There are several reports available over the high antioxidant value of seaweeds and its related pharmacological potential. Unique chemical profile of marine algae such as polysaccharides, amino acids and other bioactive compounds has diverse biological activities [6]. Moreover recent studies revealed that water soluble polysaccharide fraction derived from marine sources helps in reducing oxidative stress. Indian Porphyra (marine seaweed) due its scientifically proven nutritional benefits, it becomes now centre of attention for several researchers [6-23, 45]. Currently numerous reports are available on Porphyra and the number of reports are increasing drastically on several database. Reports also confirmed their considerable pharmacological uses such as antioxidant, anti-inflammatory and analgesic properties [6-23, 45]. Present work is designed to explore the therapeutic potential of *P. vietnamensis* [6-23, 45], to further establish a direct relation between its bioactive chemical compounds and biological activities. Due to multiple active chemical components this seaweed has tremendous nutraceutical and pharmaceutical potentials. Recent hypothesis also focused on the Porphyra components that participate in antinociceptive activity. Identification of secondary metabolites responsible for antinociceptive and antioxidant activities is again major challenge, however different porphyra extracts (PE) can be investigated to check their relative antinociceptive and antioxidant among themselves. This study was anticipated to evaluate the antinociceptive and antioxidant potential of different extracts derived from macroscopic red alga, Porphyra vietnamneis and the possible mechanisms of action of bioactive components of the extract was investigated in an *in vitro* assay [6-23, 45].

## 2. Materials and methods

### 2.1. Drugs and chemicals

Sodium nitroprusside, sulphanilamide, N-(l-naphthyl) ethylene diamine dihydrochloride, potassium ferricyanide, thiobarbituric acid, potassium nitrite, and ferric chloride were obtained from Sigma chemicals. Dypirone (Sigma Chemical), Acetic acid (Merck), Tween 20 (Sigma Chemical), indomethacin (Merck), morphine sulfate (Dimorf-Cristalia-BR), aluminium chloride (Al- Cl3), riboflavin and deoxyribose, ethylenediamine tetraacetic acid (EDTA), indianitro blue tetrazolium, sulfanilamide, naphthyleth- ylenediamine dihydrochloride (NED), thiobarbituric acid (TBA), reduced nicotinamide adenine dinucleotide (NADH), and sodium nitroprusside (SNP) were purchased from HiMedia Laboratories Pvt., Ltd, India. Dipyrone and indomethacin were used as reference drugs. Formalin solution (2.5%) was prepared by using formaldehyde (Merck) in saline solution at concentration of 0.9%. In all methods tween 80 (s.q.f.) were used and administered orally at a dose of 100 mg/kg. Dipyrone and indomethacin both were administered by oral route.

### 2.2. Preparation of extracts

For fraction preparation algal material was dried (sun drying) for almost 24 h followed by pulverization to convert into powder form and finally packed into plastic bags. Almost ten gram powdered algal material was mixed with methanol in 500 *ml* volumetric flask followed by the sonication for 4 min for 10 min. Extracts were dried further by evaporating the residual solvent. At last extracts were retreated with suitable quantity of solvents such as acetone, ethanol, chloroform and distilled water to obtain the respective extracts. From these fractions respective dilutions were made.

### 2.3. Animals

Healthy male and female Swiss mice of weight range 15-20 g of about same age group were used for antinociceptive studies. They were grouped into six groups (n = 6) of 6 animals in each group which were kept in a clean polypropylene cage and fresh drinking water was provided to them daily ad libitum. Optimal conditions were maintained at a controlled temperature (22 ± 2°C) for 12-hours light/dark cycle. The experimental studies were carried out in accordance with the guidelines of Animal Ethics Committee (Institutional Ethics Committee-IAEC/ABMRCP/2016- 2017/11). Different division of groups is mentioned in Table 1.

**Table 1:** Different groups of Swiss mice for antinociceptive studies.

Group name	Group details	Description of group
Group I	Control group	Tween 20 (1%) vehicle group was administered

Group II	Test group	Ethanolic (EE) at 100 mg/kg (p.o.) Group III
	Test group	Acetone (ACE) at 100 mg/kg (p.o.) Group IV
	Test group	Aqueous (AE) at 100 mg/kg (p.o.) Group V

Group VI	Test group	Chloroform (CE) at 100 mg/kg (p.o.)
	Test group	Dipyrone (40 mg/kg) in acetic acid-induced writhing test and indomethacin (10 mg/kg; p.o.) in hot plate method and indomethacin

### 2.4. Acetic acid-induced writhing response

Procedure introduced by Koster *et al.* [24] was followed in this experiment. Six groups (n = 6) of animals mice were made in which Group II-V administered with 100 mg/kg (p.o.) whereas Group VI was treated with 40 mg/kg of standard drug, dipyrone (p.o.). Treatment of Group I with vehicle (p.o.) was done before one hour administration of acetic acid. Acetic acid-induced writhing response was investigated for test and standard samples against the control group. Selected animals were administered with 0.6% acetic acid (IP administration) solution to get writhing response. Animals were kept in glass transparent cylinder to check the writing response accurately. Number of writings was monitored and calculated properly at 20 min intervals after the administration of acetic acid. Antinociceptive potential was evaluated by calculating percent inhibition of writhing count (treated group) from the mean count of writhing response observed in control animals.

### 2.5. Hot plate test

Eddy and Leimbach method was followed to perform this procedure [25]. PE (EE, CE, AE and ACE) at 100 mg/kg, indomethacin at 10 mg/kg and vehicle (0.5 *ml)* were administered by oral route to various groups of animals (n = 6). Eddy’s hot plate (Biotechnics India), was used as the equipment which was maintained at 55 ± 1°C. After maintaining such temperature, group wise, mice were placed on the Eddy’s hot plate. Licking of the fore and hind paws or jumping was considered as examination parameters at 0.5, 1, 1.3 and 2 h. These observations were made after the reaction time i.e. after administration of the extracts, standard drug and vehicle. The reaction time was time starts in between transferring of mice over hot plate and manifestation of signs such as licking of the fore and hind paws or jumping an effort to get away from the pain.

### 2.6. Formalin-induced nociception

We have followed protocol which was introduced by the Huns- kaar, Hole and Tjelsen *et al.* 26, 27.

Adult albino mice were randomly divided into six groups of six animals each (n = 6). 20 ^L (2.5%) of a formalin solution was administered to animals sub-plantarly in 1 hind paw. Time period of paw licking as index of nociception was monitored at 0-5 min (neurogenic phase or early phase) and from 15 to 30 min (inflammatory phase or late phase). Additionally the time that at which they start licking the injected paw was documented and calculated as indicative of nociception. PE (as prepared in above method) was administered (100 mg/kg, p.o.). One group was treated with indomethacin at 35.7 mg/kg, which was administered by oral route forty minutes before formalin administration whereas control group was only treated with prepared vehicle from Tween 20.

### 2.7. Antioxidant assays

#### 2.7.1. Total phenolic content (TPC)

Total phenolic content (TPC) of the PE was measured by using Folin-Ciocalteu protocol in which phenolic compounds form a blue complex. Gallic acid is used as a standard compound [28, 29]. During this estimation, 0.5 *ml* of Folin-Ciocalteu reagent was added to 0.1 ^l of extracts (100 ^g/ml) followed by the addition of 1 *ml* of 20% Na_2_CO_3_ solution and incubated for 10 min at room temperature. Against blank, the absorbance of reaction mixture was measured at 730 nm. The total phenolic content was expressed as gallic acid equivalents (GAE) in milligrams per gram samples.

### 2.8. DPPH antioxidant assay

DPPH is a chemical compound which is utilized in the DPPH assay to measure the capability of antioxidant compounds to suppress the concentration of DPPH radical (dark purple) by converting it into non radical form (colorless). The radical scavenging potential was determined by using DPPH [30]. This has been considered as a fundamental approach for determining the antioxidant activity of the natural products. During this process antioxidant potential of given fraction was determined by method as followed by Mahakunakorn *et al*., 2004.

### 2.9. Nitric oxide (NO) antioxidant assay

We have followed procedure which was introduced by Green, *et al.* 1982 [31]. Here in this procedure. Antioxidant principles present in the PE reduced production of NO. SNP (5 mM) in phosphate buffer saline (PBS) was mixed with different concentration of extracts (100-500 ^g/ml) and incubated at 25°C for 150 min. PE were reacted with Greiss reagent to affect the final absorbance of chromophore created during diazotization of nitrite with sul- phanilamide [31].

### 2.10. Superoxide anion (O2) antioxidant assay

Measurement of superoxide anion (O2) scavenging activity of PE was based on the method described with slight modification [32, 33]. O2 radicals are generated non enzymatically in Phenazine methosulphate-nicotinamide adenine dinucleotide phosphate (PMS-NADH) systems by the oxidation of NADH and assayed by the reduction of NBT. In this experiment, the superoxide radicals were generated in 1 *ml* of Tris-HCl buffer (16 mM, pH 8.0) containing NBT (50 ^M) solution and NADH (78 ^M) solution. The reaction was started by adding PMS solution (10 ^M) to the mixture. The reaction mixture was incubated at 25°C for 5 min, and the absorbance at 560 nm in a spectrophotometer was measured against blank samples. Decreased absorbance of the reaction mixture indicated increased superoxide anion scavenging activity. The percentage inhibition of superoxide anion generation was calculated using the following formula:

(%) I = (A0 - A1) / (A0) × 100

where A0 was the absorbance of the control and A1 was the absorbance of extract and the standard compound.

### 2.11. Hydroxyl radical (OH) scavenging activity

The scavenging activity of the different extracts on hydroxyl radical was measured according to a previously described method [30]. In 1.5 *ml* of each diluted extract, 60 ^l of FeCl_3_ (1 mmol/l), 90 ^l of 1, 10-phenanthroline (1 mmol/l), 2.4 *ml* of 0.2 mol/l phosphate buffer, pH 7.8 and 150 ^L of H_2_O_2_ (0.17 mol/L) were added respectively. The mixture was then homogenized and incubated at room temperature for 5 min. The absorbance was read at 560 nm against the blank. The percentage of the hydroxyl radical scavenging activity of each extract was calculated from the equation below:

Percentage of hydroxyl radical scavenging activity= [(OD control-OD sample)/OD control] × 100

Where OD is the optical density.

The extract concentration providing 50% inhibition (IC50) was calculated and obtained by interpolation from linear regression analysis.

### 2.12. Hydrogen peroxide (H_2_O_2_) antioxidant assay

Radical scavenging potential of prepared Porphyra extracts (PE) was determined by using H_2_O_2_ solution. At pH 7.4 given amount of H_2_O_2_ solution was prepared in phosphate buffer solution (PBS). Porphyra extract (100-500 ^ g/ml) in distilled water were added to hydrogen peroxide solution (0.6 *ml,* 40 mM). Using UV-vis spectroscopy absorbance of extract supplemented H_2_O_2_ solution was determined at 230 nm. In this step blank PBS solution (without H_2_O_2_) was used against extract supplemented H_2_O_2_ solution [34].

### 2.13. Lipid peroxidation assay

Thuong *et al.* (2007) was adopted to evaluate the antioxidant potential of PE. In his study, he has evaluated antioxidant activity of kudingcha, a traditional Chinese medicine against mitochondrial lipid peroxidation. During his work, he has used degradation or end products of lipid called as thiobarbituric acid reactive substance. These substances can be utilized determine lipid peroxidation in biological tissue [35].

### 2.14. Statistical analysis

Experimental results were mean ±SEM of three parallel measurements. Analysis of variance was performed by ANOVA followed by Newmans-Keul multiple comparison test. *p* < 0.05 was considered as significant.

## 3. Results and Discussion Antioxidant assays

### 3.1. Phenolic content estimation

**Fig. 1:**
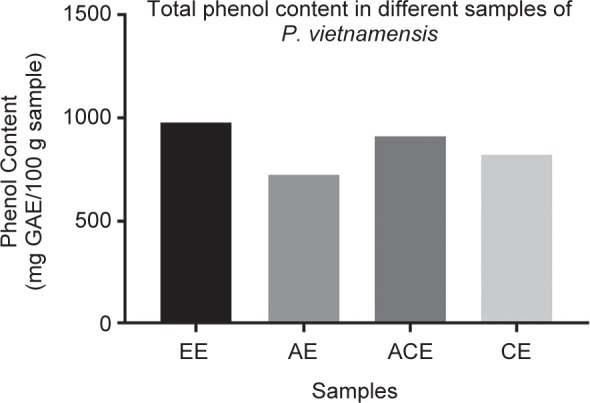
Total phenolic content analysis of different extracts derived from P *vietnamensis* Alcoholic extract (EE), Aqueous Extract (AE), Acetone extract (ACE), Chloroform extract. (CE)

Most of the plants may have diverse phytochemicals especially antioxidants which builds their antioxidant strength. This over all antioxidant profile of plant is directly proportional to the number of antioxidant principles such as polyphenols, carotenoids, and vitamins C and E present in it. Major class of the antioxidants include category of polyphenols such as flavonoids [36]. These antioxidant molecules are always versatile roles such as they also behave as anti- inflammatory, anticancer etc. Thus it’s essential to determine their total content of polyphenols present in the plant. As mentioned in Fig. 1, polyphenolic content was evaluated in all extracts was found to be in the range of 771-977 mg GAE/100g dry sample. Polyphenolic range in the given fraction was 977.0 mg GAE/gm DW. Thus in comparison with acetone, chloroform and aqueous extracts (913, 823 & 721 mg GAE /gm DW) the overall phenolic content was found to be highest in ethanolic extract (977 mg GAE /gm DW) (Fig. 1). This was little unexpected as biological activity of acetone fraction is comparatively better than other fractions.

### 3.2. DPPH assay

**Fig. 2:**
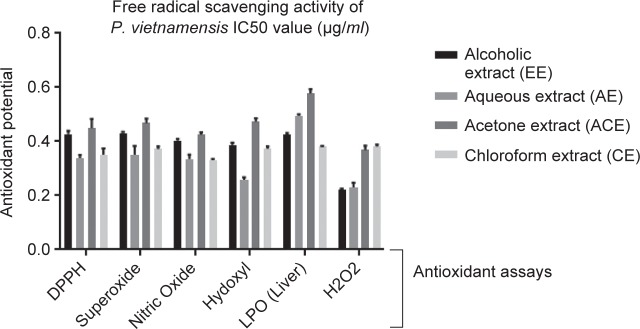
Evaluation of antioxidant potential of different extracts derived from *P vietnamensis* Alcoholic extract (EE), Aqueous Extract (AE), Acetone extract (ACE), Chloroform extract. (CE)

Underlying mechanism behind the antioxidant reaction of antioxidants molecules present in extract with free radicals (DPPH radical; purple color) produce colorless complex (a-a-diphenyl- P-picrylhydrazyl) considered as a basic and primary evaluation parameter to estimate free radical scavenging effect of the extract or any phytoconstituent. This can be examined by showing characteristic absorbance maxima at 517 nm [37]. Previous reports advocated the direct relationship between antioxidant strength and overall phenolic concentration. Phenolic compounds concentration within the isolated fractions always determines its antioxidant potential [38–40]. In current study, all PEs considerably have shown antioxidant activity against the control, which directly proportional to the concentration of phenolic content. Acetone extract have shown more significant results than other fractions (IC50 0.470 ug/ml) as depicted in Fig. 2.

### 3.3. Nitric oxide (NO) assay

Breakdown of sodium nitroprusside in a physiological solution liberate nitric oxide which can be further quenched by the phytochemical. Thus quenching or inhibition of liberated NO by different extracts is considered as a standard parameter to evaluate the antioxidant potential of respective compounds/fractions. Reaction between reactive oxygen species (ROS) and NO always results in the production of reactive nitrogen species (RNS). This category of RNS includes free radicals such as NO_2_, N_2_O_4_ *etc.* These free radicals always results in severe cellular injury which can be prevented by treatment with different antioxidant compounds [41]. In the present work different fractions significantly decreased the release of NO in a dependent manner. Here in this investigation, radical scavenging activity potential of different fractions derived from *P. vietnamensis* increased up to 70% at the concentration of 0.434 ug/ml. Nitrite oxide radical scavenging potential of these acetone fractions was found to be higher than aqueous and alcoholic extracts. Other extracts such as AE, CE and EE exhibited moderated or weak scavenging activity whereas all algal extracts showed significant scavenging activity in comparison to the control (Fig. 2).

### 3.4. Superoxide radical scavenging assay

This assay involves decomposition reaction triggered by superoxide radical 42. This decomposition reaction determines by several reactions. Current investigation suggested role of different extracts derived from *P. vietnamensis* in neutralizing superoxide radicals. In phenazine methosulfate/NADH-nitro blue tetrazo- lium system the reduction of NBT with NADH mediated through PMS was suppressed by the addition of 50% acetone fraction (ACE) from *P. vietnamensis*. Superoxide anion radical scavenging potential of acetone extracts (ACE) determines by evaluating the effectiveness of extract in reducing formazan formation. Thus extracts would consequently reduces the steady-state concentration of NBTH by scavenging O2^-^, resulting in the decline rate of the production of formazan. Therefore, the shift in absorbance (570 nm) due to the presence of antioxidants resulted in the reduction of the concentration of formazan dye. PAE has shown considerable antioxidant activity (IC50 0.470 ug/ml) (Fig. 2).

### 3.5. Hydrogen peroxide (H2O2) radical assay

H_2_O_2_ liberated radicals are often not very reactive however it cause cellular injury as it produces OH radical in the cell [43]. It has been observed that acetone extract considerably inhibited peroxide radical with IC50 value 0.381 ug/ml (Fig. 2).

### 3.6. Lipid peroxidation assay

It has been reported that lipid breakdown resulted in generation of end products such as malondialdehyde, aldehydes *etc*. Reaction between end products and thiobarbituric acid reactive substances resulted in the formation of malondialdehyde- thiobarbituric acid reactive substances complex. This colorful complex was deter

mined by spectrophotometeric analysis by UV spectrophotometer at 532 nm. Because of the presence of porphyran, aqueous fraction showed highest anti-lipid peroxidation effect. This effect was observed in liver homogenate with IC50 value of 0.591 ^g/ *ml* (Fig. 2). These results demonstrated that inhibition of TBARS formation in rat liver homogenate increased with increasing concentration of porphyran [44].

### 3.7. Hydroxyl radical assay

OH one of the well known reactive oxygen species which are produced as by-products of cellular metabolism and considered as one of the most reactive species which can cause severe oxidative injury to macromolecules. Antioxidant potential of PEs was investigated by iron (II) dependent DNA damage assay. In comparison with others, acetone extract has shown considerable inhibition (IC50 0.477 ^g/ml) (Fig. 2).

### 3.8. Antinociceptive potential of extracts

The antinociceptive activity of extracts derived from Porphyra was evaluated by using murine pain models, namely acetic acid- induced writhing, hot plate and formalin-induced nociception tests.

### 3.9. Acetic acid-induced writhing

**Fig. 3:**
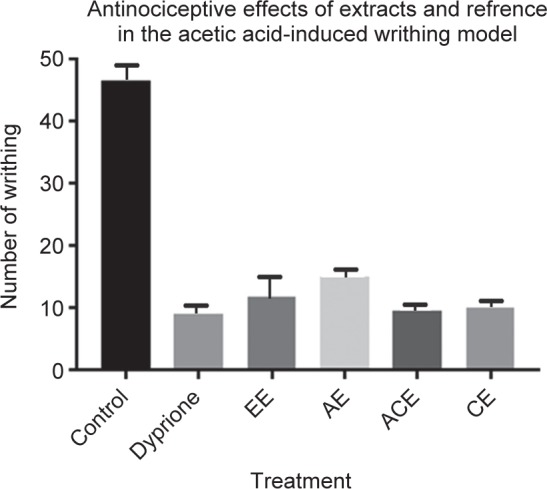
Antinociceptive potential of Porphyra extracts Alcoholic extract (EE), Aqueous Extract (AE), Acetone extract (ACE), Chloroform extract. (CE)

Ethanolic (EE), acetone (ACE), aqueous (AE) and chloroform (CE) extracts (at a dose of 100 mg/kg) derived from Porphyra genus were pre administered to respective groups of animals. This pretreatment of animals with different extracts causes considerable inhibition of the acetic acid- induced writhing response. All extracts (EE, ACE, AE CE extracts) considered for the study showed high peripheral antinociceptive activity with an inhibition of 88.1%, 91.5%, 84.8% and 89.9.0%, respectively. Furthermore, these positive results in the writhing test were also observed for dipyrone (86.5%), as expected, used as the reference peripheral analgesic drug (Fig. 3). These reports advocate that there is a possible antinociceptive action for extracts derived from genus Porphyra.

### 3.10. Hot plate method

Results of the Porphyra extracts in the hot plate method were demonstrated in Table 2. thermal nociception test was performed to evaluate antinociceptive potential of Porphyra extracts. In this method, the aqueous fraction (AE) did not considerably increase the latency of response. This showed that they do not exhibit central activity. In contrast, administration of EE, AE, ACE and CE resulted in the significant increase in the latency time of the animals especially in the case of ethanolic (EE) and acetone (ACE) fractions. Findings suggested that acetone fraction showed considerable analgesic activity in animal model however chloroform (CE) and aqueous fractions (AE) have not shown considerable

**Table 2:** Treatment of Porphyra extracts and Indomethacin in hot plate test. (thermal nociception)

After administration of Porphyra extracts (100 mg/kg, p.o.) against control and standard drug (Indomethacin 10 mg/kg, p.o) in hot plate test.					
Groups	Time after injection. (min)				
	0	30	60	90	120
Control	1.1 ± 0.2	1.5 ± 0.4	2.1 ± 0.6	2.6 ± 0.1	3.4 ± 0.4
Indomethacin	4.5 ± 0.3	5.3 ± 0.2	9.1 ±	7.8 ± 0.2	9.7 ± 0.3
			0.1**		
EE	1.7 ± 0.3	2.5 ± 0.2	3.1 ± 0.1	4.6 ± 0.4	5.2 ± 0.2
AE	1.3 ± 0.2	1.7 ± 0.1	2.9 ± 0.3	3.7 ± 0.2	3.4 ± 0.1**
ACE	1.9 ± 0.1	2.7 ± 0.3	4.1 ± 0.5	5.1 ± 0.1	6.2 ± 0.4
CE	1.4 ± 0.2	2.3 ± 0.4	2.9 ± 0.3	3.4 ± 0.2	4.3 ± 0.1

analgesic effect in hot plate test. This demonstrates cause behind the significant analgesic activity for these extracts which could be arbitrated by mean of the suppression of pain receptors or mediator’s synthesis suppression of cyclooxigenase. In the hot plate test indomethacin administration (5 mg/kg, s.c.), resulted in the considerable increase in latency time, which last for not less than 150 min. Indomethacin (NSAIDs) was used as standard drug for inducing peripheral analgesic properties as it caused a significantly suppression in pain threshold at (5 mg/kg, s.c.).

### 3.11. Formalin test

**Fig. 4:**
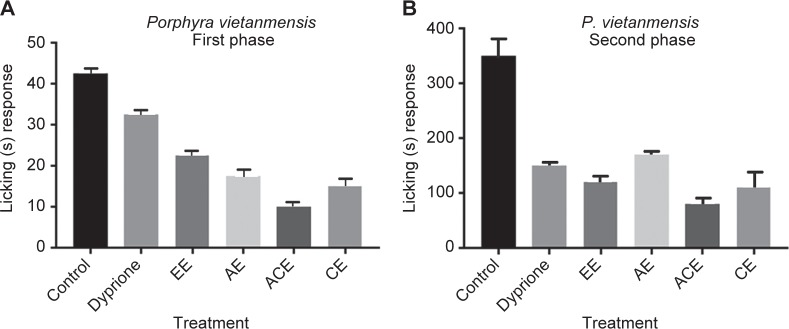
4A Licking response during first phase; 4B Licking response during second phase Alcoholic extract (EE), Aqueous Extract (AE), Acetone extract (ACE), Chloroform extract. (CE)

Formalin test is reliable model to evaluate the neurogenic and inflammatory pain. In the current study it has been utilized to evaluate the effects of analgesic compounds in mouse or rat. Formalin was injected into hind paw to induce biphasic pain response *i.e*. first phase related with acute neurogenic pain, while the second phase related with inflammatory pain. This period of biphasic pain last for 60 min. Administration of different extracts such as EE, ACE, AE and CE from *P. vietnemensis* resulted in the inhibition of 55%, 80%, 65% and 70%, respectively; in the first phase (Fig. 4A). In addition, all extracts of *P. vietnemensis* reduced inflammation significantly in the second phase, with an inhibition of 65%, 90%, 71% and 70%, (Fig. 4B). It has been observed that acetone fraction showed significant anti-inflammatory activity which was even better than the standard drug (indometha- cin). However it wasn’t true for all of the extracts as most of the extracts reduced formalin induced nociception in the second phase, but failed during first initial phase. Since anti-inflammatory potential of all the fractions was observed in both phases of formalin-induced edema thus pharmacological investigation revealed confirmed the antinociceptive potential of the extracts. Possible cause behind the inhibition could be suppression of the production of several mediators such as prostaglandins.

## 4. Conclusion

Several red algae around the world have been extensively investigated by the researchers for their several applications. Most of the Asian countries include this alga in their routine diet as “Sushi” Nevertheless consumption and cultivation of edible algae is very less India and most of the national are not familiar with their importance. Thus current research is aimed to explore the therapeutic compounds present in the isolated fraction. As most of the seaweed contains extensive range of anti-oxidative compounds, these active principle can utilized for any other therapeutic ailment. Several reports on the radical scavenging potential of the components are available however the comparative potential of different fractions derived from *P. vietnamensis* is unavailable. Here in this study we have done comparative analysis of *P. viet- namensis* and discovered that out of different fractions acetone fraction showed considerable antioxidant activity. Similarly while determining antinociceptive potential of different fractions derived from *P. vietnamensis* it has been observed that acetone fraction represents antinociceptive potential predominantly then others. It has been also noticed that all /manner. It can be argued that the extracts exhibit antinociceptive potential and may be due to antioxidant potential. Nevertheless, further investigation is required to characterize the mechanism(s) responsible for the antinociceptive action and identify agents that are responsible for this activity in alga.

## References

[1] Kumar R , Zi-Rong X . Biomedical Compounds from Marine organisms. Mar Drugs. 2004; 2(1): 123–46

[2] Nisizawa K , Noda H , Kikuchi R , Watamaba T . The main seaweed foods in Japan. Hydrobiol. J., v. 1987; 151/152(1): 5–29

[3] Faulkner DJ . Marine natural products. Nat Prod Rep. 2002; 19: 1–48 11902436 10.1039/b009029h

[4] Smit AJ . Medicinal and pharmaceutical uses of seaweed natural products: a review. J. Appl. Phycol. 2004; 16: 245–62

[5] Burtin P . Nutritional value of seaweeds. EJEAF Che. 2003; 2: 498503

[6] Bhatia S , Namdeo AG , Nanda, S . Factors effecting the gelling and emulsifying properties of a natural polymer. Syst Rev Pharm. 2010b; 1(1): 86–92

[7] Bhatia S , Garg A , Sharma K , Kumar S , Sharma A , Purohit AP . Mycosporine and mycosporine-like amino acids: A paramount tool against ultra violet irradiation. Pharmacog Rev. 2011; 5(10): 138–46 10.4103/0973-7847.91107 PMC3263047 22279371

[8] Bhatia S , Rathee P , Sharma K , Chaugule BB , Kar N , Bera T . Immuno-modulation effect of sulphated polysaccharide (porphyran) from Porphyra vietnamensis. Int. J. Biol. Macromol. 2013; 57: 50–6 23500431 10.1016/j.ijbiomac.2013.03.012

[9] Bhatia, S. , Sharma, A. , Sharma, K. , Kavale, M. , Chaugule, B.B. , Dhalwal, K. , et al., Novel Algal Polysaccharides from Marine Source: Porphyran. Pharmacognosy Review. 2008; 2(4): 271–6

[10] Bhatia, S. , Sharma, K. , Namdeo, A.G. , Chaugule, B.B. , Kavale, M. , Nanda, S. Broad-spectrum sun-protective action of Porphyra-334 derived from Porphyra vietnamensis. Pharmacog. Res. 2010a; 2(1): 45–9 10.4103/0974-8490.60578 PMC3140129 21808539

[11] Bhatia S , Kumar V , Sharma K , Nagpal K , Bera T . Significance of Algal Polymer in Designing Amphotericin B Nanoparticles. The Scientific World J. 2014, Article ID 564573, 21 10.1155/2014/564573 PMC4244925 25478596

[12] Bhatia S , Sharma K , Nagpal K , Bera T . Investigation of the factors influencing the molecular weight of porphyran and its associated antifungal activity. Bioactive Carbohydrates and Dietary Fibre. 2015a; 5(2): 153–68

[13] Bhatia S , Sharma K , Sharma A , Nagpal K , Bera T . Anti-inflammatory, Analgesic and Antiulcer properties of Porphyra vietnamensis. Avicenna J Phytomed. 2015b; 5(1): 69–77 25767759 PMC4352535

[14] Bhatia S , Sharma K , Bera T . Structural characterization and pharmaceutical properties of porphyran. Asian J Pharm. 2015; 9: 93–101

[15] Bhatia S. , Bera T. Evaluation of pharmacognostical, phytochemical and anti-microbial properties of Porphyra vietnamensis. International Journal of Green Pharmacy. 9(2); 2015c: 131–7

[16] Bhatia S , Sharma K , Sharma A , Namdeo AG , Chaugule BB . Antioxidant potential of Indian porphyra. Pharmacologyonline 2011; 1: 248–57

[17] Bhatia S , Sharma K , Dahiya R , Bera T . Modern Applications of Plant Biotechnology in Pharmaceutical Sciences. Academic press, Elsevier 2015d; 164–74

[18] Bhatia S . Nanotechnology in Drug Delivery: Fundamentals, Design, and Applications. CRC press 2016; 121–7

[19] Bhatia S , Goli D . Leishmaniasis: Biology, Control and New Approaches for Its Treatment. CRC press 2016a; 164–73

[20] Bhatia S . Natural Polymer Drug Delivery Systems: Nanoparticles, Plants, and Algae, Springer Nature 2016b; 117–27

[21] Bhatia S . Systems for Drug Delivery: Safety, Animal, and Microbial Polysaccharides, Springer Nature 2016c; 122–7

[22] Bhatia S . Introduction to pharmaceutical biotechnology, 1st Vol, IOP Publishing house Bristol 2018a; 167–74

[23] Bhatia S . Introduction to Pharmaceutical Biotechnology, 2nd vol, IOP publishing house Bristol 2018b; 172–77

[24] Koster R , Anderson M , De-Beer E.J. Acetic acid analgesic screen. Fed Proc. 1959; 18: 418–20

[25] Eddy N.B , Leimbach D . Synthetic analgesics. II. Dithienylbutenyl and dithienylbutylamines. J Pharmacol Exp Ther. 1953; 107: 385–93 13035677

[26] Hunskaar S. , Hole K. The formalin test in mice: dissociation between inflammatory and non-inflammatory pain. Pain. 1987; 30: 103–14 3614974 10.1016/0304-3959(87)90088-1

[27] Tjolsen A , Hole K. Animal models of analgesia. In The Pharmacology of Pain. Dickenson, A. , Besson, J. , Eds.; Springer Verlag: Berlin, Germany, 1997; 130: 1–20

[28] Harborne JB . Methods of Extraction and Isolation. In: Phytochemical Methods. London, Chapman and Hall, 1998:60–66

[29] Kujala TS , Loponen JM , Klika KD , Pihlaja K . Phenolic and beta- cyanins in red beet root (Beta vulgaris) root: distribution and effects of cold storage on the content of total phenolics and three individual compounds. J Agric Food Chem. 2000; 48: 5338–42 11087483 10.1021/jf000523q

[30] Mahakunakorn P , Tohda M , Murakami Y , Matsumoto K , Watanabe H . Antioxidant and free radical scavenging activity of chitosan and its related constituents. Biol Pharm Bull. 2004; 27: 38–46 14709896 10.1248/bpb.27.38

[31] Green LC , Wagner DA , Glogowski J . Analysis of nitrate, nitrite and 15 (N) nitrate in biological fluids. Anal Biochem. 1982; 126: 131–8 7181105 10.1016/0003-2697(82)90118-x

[32] Liu F , Ooi VEC , Chang ST . Free radical scavenging activity of mushroom polysaccharide extracts. Life Sci. 1997; 60: 763–71 9064481 10.1016/s0024-3205(97)00004-0

[33] Oktay M , Gulcin I , Kufrevioglu OI . Determination of in vitro antioxidant activity of fennel (Foeniculum vulgare) seed extracts. Lebensmittel Wissenchaft und Technol. 2003; 36: 263–71

[34] Ruch RJ , Cheng SJ , Klaunig JE . Prevention of cytotoxicity and inhibition of intracellular communication by antioxidant catechins isolated from Chinese green tea. Carcinogen. 1989; 10: 1003–8 10.1093/carcin/10.6.1003 2470525

[35] Thuong PT , Kang HJ , Na MK , Jin WY , Youn UJ , Seong YH . Antioxidant constituents from Sedum takesimense. Phytochem. 2007; 68: 2432–8 10.1016/j.phytochem.2007.05.031 17658562

[36] Yen GC , Duh PD , Tsai CL . The relationship between antioxidant activity and maturity of peanut hulls. J Agric Food Chem. 1993; 41: 67–70

[37] Jao CH , Ko WC . 1, 1-Diphenyl-2-picrylhydrazyl (DPPH) radical scavenging by protein hydrolyzates from tuna cooking juice. Fish Sci. 2002; 68: 430–5

[38] Oki T , Masuda M , Furuta S , Nishiba Y , Terahara N , Suda I . Involvement of anthocyanins and other phenolic compounds in radical scavenging activity of purple fleshed sweet potato cultivars. Food Chem Toxicol. 2002; 67: 1752–6

[39] Lu Y , Foo YL . Antioxidant and free radical scavenging activities of selected medicinal herbs. J Life Sci. 2000; 66: 725–35 10.1016/s0024-3205(99)00643-8 10680580

[40] Siriwardhana N , Lee KW , Kim SH , Ha JW , Jeon YJ . Antioxidant activity of Hizikia fusiformis on reactive oxygen species scavenging and lipid peroxidation inhibition. Food Sci Tech Int. 2003; 9: 339–46

[41] Pacifici RE , Davies KJ . Protein, lipid and DNA repair system in oxidative stress: The free radical theory of aging revisited. Gerontol. 1991; 37:166–80 10.1159/000213257 2055497

[42] Dahl M , Richardson M . Photogeneration of superoxide anion in serum of bovine milk and in model systems containing riboflavin and amino acids. J Dairy Sci. 1978; 61: 400–7

[43] Halliwell B , Gutteridge JMC , Amoma OL . The deoxyribose method, a simple test tube assay for the determination of rate constant for reactions of hydroxyl radicals. Anal Biochem. 1987; 165: 215–9 3120621 10.1016/0003-2697(87)90222-3

[44] Nohl H. Involvement of free radicals in ageing: A consequence or cause of senescence. Brit Med Bull. 1993; 49: 653–67 8221030 10.1093/oxfordjournals.bmb.a072638

[45] Bhatia S , Goli D , Naved T , Sharma A . Nutraceutical Properties of Indian Seaweed Porphyra. Adv Inv Pharmacol Therapeutic Med. 2018; 1: 47–54

